# A Single-Center Retrospective Study on the Clinical Outcomes of TightRope Fixation Versus Syndesmotic Screw Fixation in the Management of Acute Traumatic Ankle Syndesmotic Injuries

**DOI:** 10.7759/cureus.76153

**Published:** 2024-12-21

**Authors:** Fang Fang Quek, Humam Jundi, Ioannis Aktselis, Mosab Elgalli

**Affiliations:** 1 Trauma and Orthopaedic Surgery, Buckinghamshire Healthcare NHS Trust, Aylesbury, GBR; 2 Trauma and Orthopaedics, Buckinghamshire Healthcare NHS Trust, Aylesbury, GBR; 3 Orthopaedic Surgery, Buckinghamshire Healthcare NHS Trust, Aylesbury, GBR

**Keywords:** ankle, fractures, surgery, syndesmosis, syndesmotic screws, tightrope

## Abstract

Background

Ankle fractures are one of the most common presentations in orthopaedic surgery and represent the third most frequent musculoskeletal injury in the elderly population. Syndesmotic injuries can be associated with ankle fractures, and surgical intervention is critical in these injuries to restore stability and prevent long-term disability. Traditionally, syndesmotic screw fixation has been the standard treatment for acute traumatic syndesmotic injuries, but controversies regarding this fixation method remain. Over recent years, the TightRope system (Arthrex, Florida, US) has gained popularity as a dynamic alternative, offering the advantage of restoring anatomical function while maintaining reduction. The optimal surgical fixation method for managing syndesmotic injuries remains a topic of ongoing debate within orthopaedic practice. Therefore, this study aims to compare the clinical outcomes of these two fixation methods to provide further guidance on their use in managing acute traumatic syndesmotic injuries.

Methods

A retrospective cohort study was performed for all patients with ankle syndesmotic injuries who underwent surgical fixation using either TightRope devices or syndesmotic screws at Buckinghamshire Healthcare NHS Trust between June 2020 and June 2023, identified through the BlueSpier electronic record system (Bluespier, Droitwich, United Kingdom). Data on demographics and surgical details were extracted from electronic medical records while radiographic images were systematically reviewed to confirm eligibility for inclusion. Clinic letters were also reviewed for complications and reasons for metalwork removal.

Results

A total of 217 patients met the eligibility criteria for this study, with 132 (61%) females and 85 (39%) males, aged between 13 and 93 years (mean age: 49 years). Of the cohort, 28 (13%) underwent syndesmotic fixation with TightRope devices while 189 (87%) were treated with syndesmotic screws. Metalwork removal was required in 11% of TightRope cases (3 patients) and 28% of syndesmotic screw cases (52 patients). The most common reason for metalwork removal in our study cohort was for broken or loosened screw(s), followed by discomfort and patient preferences. The metalwork removal rates in our study cohort are consistent with those reported in the current literature.

Conclusion

In conclusion, our study found that the use of TightRope devices is associated with lower removal rates in comparison to syndesmotic screws. This finding is consistent with those reported in the current literature. The most common documented reason for metalwork removal in our study cohort was due to screw breakage or loosening. Although emerging evidence suggests that routine removal of syndesmotic screws may not be necessary, given the lack of consensus regarding the routine removal of syndesmotic screws, decisions for metalwork removal should be tailored by clinical judgement and individual patient needs. Despite its limitations, this study contributes valuable insights into the outcomes and metalwork removal rates associated with syndesmotic fixation methods in the management of acute ankle fractures with syndesmotic injuries.

## Introduction

Ankle fractures are one of the most common presentations in orthopaedic surgery, constituting approximately 10% of all reported bony injuries [1]. They remain the third most common musculoskeletal injury in the elderly population, with recent studies reporting a rising incidence and severity of ankle fractures in this population [2,3]. Among all reported ankle fractures, syndesmotic injuries are present in 10% to 13% of cases [4]. Syndesmotic instability is a characteristic feature of all Weber C fractures and approximately half of all Weber B fractures [5]. Timely surgical intervention is crucial in these cases to restore stability and function and to mitigate the risks of long-term disability [6].

Traditionally, syndesmotic fixation using syndesmotic screws, as recommended by the AO group, has been the gold standard in the management of syndesmotic injuries [7]. This approach involves inserting one or more syndesmotic screws through the fibula into the tibia to stabilise the syndesmosis to ensure structural integrity [8]. However, despite its widespread use, there remain several controversies related to syndesmotic screw fixation, including the optimal number of cortices required for stabilisation, the ideal level of screw placement above the tibial plafond, the appropriate screw size, and the necessity and timing of screw removal [9-11]. In recent years, the introduction of the TightRopeTM system (Arthrex, Florida, US) has provided a novel alternative. This implant, based on a suture endobutton design, consists of a non-absorbable synthetic suture that connects two metallic buttons placed across the syndesmosis, effectively restoring its anatomic function while maintaining reduction [4].

The optimal surgical fixation method for managing ankle fractures with associated syndesmotic injury, whether to use TightRope devices or syndesmotic screws, remains a topic of ongoing debate within the orthopaedic practice. Therefore, this study aims to provide a comprehensive analysis by comparing the clinical outcomes between both fixation methods in our Trust to provide further guidance on their use in managing syndesmotic ankle fractures.

## Materials and methods

Patients and methods

A retrospective cohort study was performed for all patients who sustained acute traumatic ankle syndesmotic injuries and underwent surgical fixation using either TightRope or syndesmotic screws at Buckinghamshire Healthcare NHS Trust, United Kingdom, between June 2020 to June 2023.

Inclusion and Exclusion Criteria

**Figure 1:**
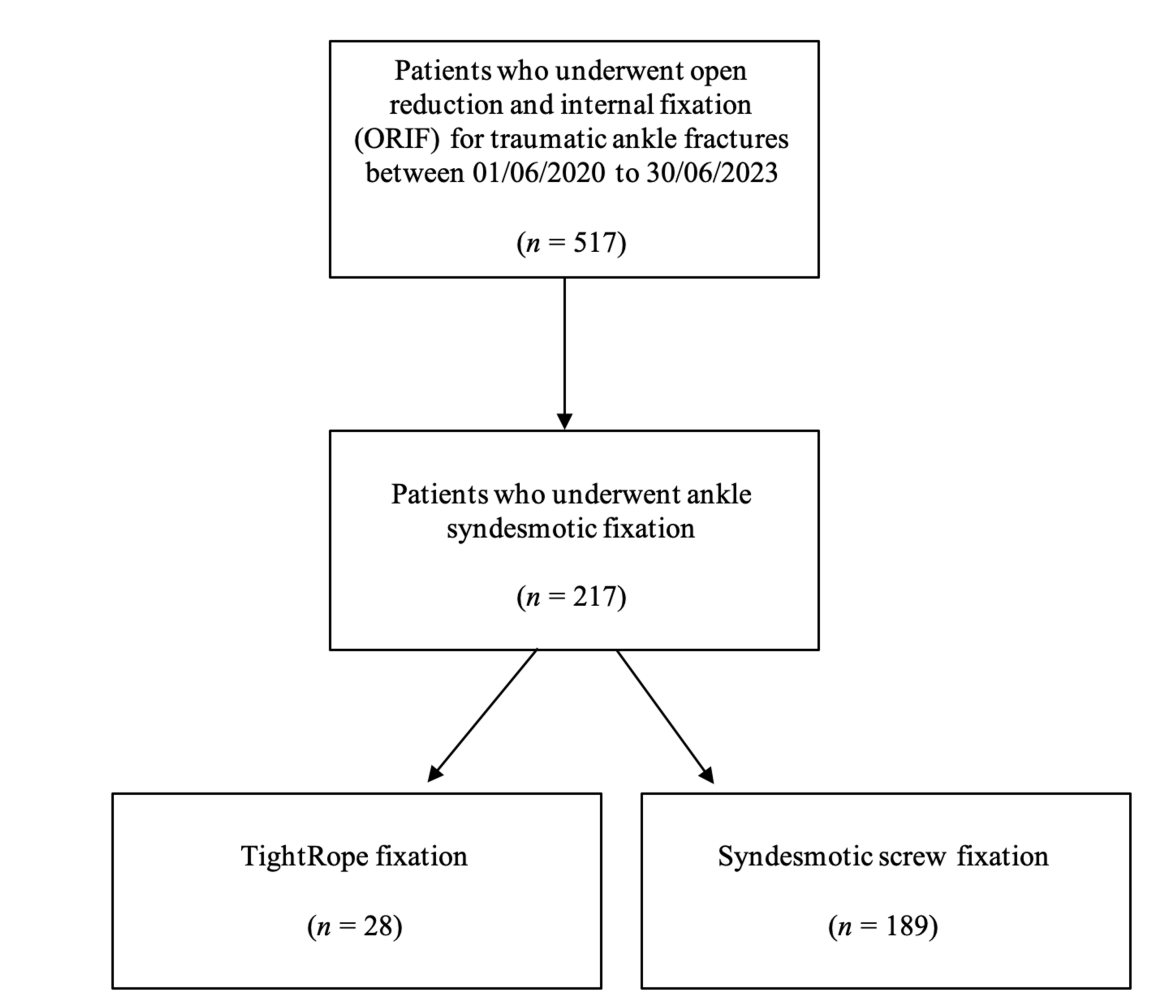
Schematic diagram illustrating the study methodology

Only data relating to patients with radiologically confirmed ankle fractures with syndesmotic injuries and subsequently underwent ankle syndesmotic fixation between June 2020 to June 2023 were considered. All patients who underwent ankle syndesmotic fixations surgeries were identified from our Trust database using the Bluespier theatre system reporting tool (Bluespier, Droitwich, United Kingdom). Patients managed non-operatively were excluded from this study. Clinical data, including demographic information, surgical details and postoperative outcomes, were collected from electronic medical records. Radiographic imaging was systematically reviewed for all patients to determine the type of surgical fixation methods and to ensure eligibility for inclusion in this study. Clinic letters were subsequently reviewed to identify any complications and subsequent or planned surgeries, particularly for the removal of metalwork. Figure 1 illustrates the detailed methodology of this study.

A retrospective cohort study was performed for all patients who sustained acute traumatic ankle syndesmotic injuries and underwent surgical fixation using either TightRope or syndesmotic screws at Buckinghamshire Healthcare NHS Trust, United Kingdom, between June 2020 and June 2023.

## Results

A total of 217 patients met the eligibility criteria for this study. Our study cohort consisted of 132 (61%) female patients and 85 (39%) male patients. In this study cohort, patients who underwent primary ankle syndesmotic fixation following traumatic ankle injury ranged in age from 13 to 93 years, with a mean age of 49 years (Table 1).

**Table 1:** Demographics and characteristics of patients (n = 217)

Characteristics	Number of patients: n (%)
Sex	
Male	85 (39)
Female	132 (61)
Age (Years)	
<50	111 (51)
50-59	44 (20)
60-69	24 (11)
70-79	26 (12)
≥80	12 (6)

Of the 217 patients included in this study, 28 (13%) underwent syndesmotic fixation using TightRope devices while 189 (87%) were treated with syndesmotic screws (Table 2).

**Table 2:** Clinical characteristics Types of syndesmotic fixation (n = 217)

Types of Syndesmotic Fixation	Number of patients: n (%)
TightRope	28 (13)
Syndesmosis Screws	189 (87)

Table 3 presents the demographics of patients who received syndesmotic screw fixation.

**Table 3:** Demographics and characteristics of patients who received syndesmotic screw fixation (n = 189)

Patient Demographics	n (%)
Sex	
Male	70 (37)
Female	119 (63)
Age (Years)	
<50	89 (47)
50-59	40 (21)
60-69	22 (12)
70-79	26 (14)
≥80	12 (6)

 Table 4 presents the demographics of patients who received TightRope fixation.

**Table 4:** Demographics and characteristics of patients who received TightRope fixation (n = 28)

Patient Demographics	n (%)
Sex	
Male	15 (54)
Female	13 (46)
Age (Years)	
<50	22 (79)
50-59	4 (14)
60-69	2 (7)
70-79	0 (0)
≥80	0 (0)

In the TightRope cohort (n = 28), 25 patients (89%) retained the implant, with only 3 patients (11%) undergoing metalwork removal (Table 5). In contrast, among those treated with syndesmotic screws (n = 189), a higher proportion required hardware removal, with 52 patients (28%) having the screws removed while 137 patients (72%) retained the implants. These findings highlight a notable difference in the rates of metalwork removal between the two fixation methods.

**Table 5:** Removal of metalwork *Information accurate up to the writing of this paper (June 2024).

Implant Type	Status	n (%)
TightRope (n = 28)	Not removed	25 (89)
	Removed	3 (11)
Syndesmosis Screws (n = 189)	Not removed	137 (72)
	Removed	52 (28)

Based on the review of clinic letters, metalwork removal was documented in 57 cases for a variety of reasons. The most commonly documented reason was broken or loosened screws, accounting for 19 cases. Pain and/or irritation related to the hardware was reported in 17 cases while removal following infection was documented in 4 cases. Additionally, in 17 cases, removal was performed due to patient preference. These findings emphasise the range of clinical factors influencing the decision to remove metalwork following syndesmotic fixation. Table 6 presents the various clinical indications for the removal of metalwork following syndesmotic fixation.

**Table 6:** Reasons for metalwork removal as documented in clinic letters This table summarises the documented reasons for metalwork removal as recorded in clinic letters. Only explicitly recorded reasons for metalwork removal in clinic letters were included. Out of all metalwork removals, reasons for removal were documented in 57 cases (30%).

Reason for Removal	n (%)
Broken screw/Loosening of screw	19 (33)
Pain/Irritation	17 (30)
Infection	4 (7)
Patient preference	17 (30)

## Discussion

Ankle fractures are one the most common presentations in orthopaedic surgery and approximately 20% of all ankle fractures require surgical intervention for their associated syndesmotic injury [12]. The distal tibiofibular syndesmosis complex is crucial in maintaining ankle mortise congruency and injury to this structure can disrupt ankle stability [13]. When syndesmotic injuries are left untreated, they can lead to chronic instability and eventual degenerative arthritis [14]. Therefore, surgical intervention is usually indicated for ankle fractures with associated syndesmotic injuries. Ankle syndesmotic fixation can be achieved either through static fixation using syndesmotic screw(s) or dynamic fixation using a suture button TightRope device [6]. The choice between TightRope and syndesmotic screw fixation is usually based on surgeon preference, reflecting individual clinical judgment and expertise in determining the most appropriate approach for each patient.

Traditionally, syndesmotic screw has been the most popular fixation method. However, studies have reported complications associated with this fixation method, including syndesmotic mal-reduction and potential late diastasis due to screw breakage or screw removal [15]. In recent years, the flexible TightRope suture-button device has been gaining popularity as the fixation method for syndesmotic instability [16]. The advantages of the TightRope device include dynamic fixation of the syndesmosis, which allows for physiological movement of the syndesmosis, lower risk of metalwork removal and the ability to commence earlier rehabilitation [15]. The optimal surgical fixation method for treating ankle fractures with a syndesmotic injury remains a subject of ongoing debate. Therefore, this study aims to address this by analysing and comparing the clinical outcomes between both fixation methods based on our experience in our Trust.

The need for routine syndesmotic removal remains controversial [17]. Despite previously being the standard of care, recent studies have not recommended the routine removal of syndesmotic screws [18]. However, reported complications associated with the retention of syndesmotic screws include a reduced range of ankle motion and persistent discomfort [19]. In our study cohort, metalwork removal was required in 11% of cases for TightRope devices and 28% of all syndesmotic screw fixation. These findings align with the rates reported in the current literature, with TightRope removal rates ranging between 0% and 10% [20]. In contrast, the removal rates for syndesmotic screws show greater variability in the literature, ranging from 5% to 52% [20]. This wide variability in the reported removal rates for syndesmotic screws is likely attributed to differing practices among surgeons and the lack of a standardised approach regarding routine removal. The most common reason for metalwork removal in our study cohort was broken or loosened screw(s), followed by discomfort and patient preferences. Although emerging evidence suggests that routine removal of syndesmotic screws may not be necessary, decisions for metalwork removal are often influenced by clinical judgement and the preferences of individual patients [21].

This retrospective cohort study has several limitations that should be considered. Firstly, the reliance on data extracted from electronic medical records may introduce reporting inconsistencies, as the accuracy of the data depends on the completeness of documentation. Additionally, as this study is limited to a single institution with a relatively small cohort of patients who underwent TightRope fixation, this may affect the generalisability of our result findings. Lastly, as some of the surgeries were recent, this may potentially underestimate the removal rates, as our study has found that most of these metalwork removals were performed at an average of 232 days after the primary procedure.

## Conclusions

In conclusion, our study found that the use of TightRope devices is associated with lower removal rates in comparison to syndesmotic screws. This finding is consistent with that reported in the current literature. The most common documented reason for metalwork removal in our study cohort was screw breakage or loosening. Although emerging evidence suggests that routine removal of syndesmotic screws may not be necessary, given the lack of consensus regarding the routine removal of syndesmotic screws, decisions for metalwork removal should be tailored by clinical judgement and individual patient needs. Despite its limitations, this study contributes valuable insights into the outcomes and metalwork removal rates associated with syndesmotic fixation methods in the management of acute ankle fractures with syndesmotic injuries.
